# G^2^ continuity conditions for generalized Bézier-like surfaces with multiple shape parameters

**DOI:** 10.1186/s13660-017-1524-7

**Published:** 2017-10-04

**Authors:** Gang Hu, Huanxin Cao, Xing Wang, Xinqiang Qin

**Affiliations:** Department of Applied Mathematics, Xi’an University of Technology, Xi’an, Shaanxi 710054 China

**Keywords:** 65D07, 65D10, 65D17, 65D18, 68U05, 68U07, Bernstein-like basis functions, generalized Bézier-like surfaces, shape parameter, G^2^ continuity conditions

## Abstract

In order to tackle the problem of shape design and shape adjustment of complex surfaces in engineering, continuity conditions between generalized Bézier-like surfaces with multiple shape parameters are studied in this paper. Firstly, the geometric model of the generalized Bézier-like surfaces is built by blending a number of Bézier-like curves with independent shape parameters. Secondly, based on the terminal properties and linear independence of Bernstein-like basis functions, the conditions for G^2^ continuity between two adjacent generalized Bézier-like surfaces are derived, and then simplified by choosing appropriate shape parameters. Finally, some properties and applications of the smooth continuity between generalized Bézier-like surfaces are discussed. The modeling examples show that the proposed method is effective and easy to implement, which can greatly improve the ability to construct complex surfaces by using the generalized Bézier-like surfaces.

## Introduction

In the practical applications, since the appearance modelings of many products in industry are quite complex, they often cannot be described by a single surface in many cases. Thus, there is a need to design such products using adjacent surfaces. The smooth continuity among multiple surface patches with certain smooth constraints is usually used to achieve the appearance design of complex products. The ultimate aim of smooth continuity is to make adjacent surface patches satisfy certain smooth conditions so that the complex piecewise surface composed of these surface patches has global smoothness visually. Parametric surfaces, which are not only the standard form for the mathematical description of product appearance in CAD/CAM, but also a powerful tool for various shape designs and geometric representations, have received much attention since the 1960s. Thus the smooth continuity between parametric surfaces is an important method to construct complex surfaces and also significant research in the CAD/CAM system [1, 2].

There are two kinds of measuring standards established for the continuity of piecewise parametric surfaces [3]: (1) parametric continuity, which is usually called $\mathrm{C}^{n}$ continuity; (2) geometric continuity, or $\mathrm{G}^{n}$ continuity for short. However, the parametric continuity of a surface is relevant to its selected parameter and is usually valid under certain ones. In addition, if the common boundary of two adjacent surfaces is irregular, even though the two surfaces satisfy C^1^ continuity at the joint, it does not necessarily mean that the two surfaces possess a common tangent plane at any point on their common boundary. That is, the piecewise surface composed of the two adjacent surfaces may not be smooth at the joint. So the smooth continuity between surfaces cannot be exactly measured only by the parametric continuity [3]. In addition, the smoothness of surfaces is a kind of geometric characteristic. Therefore, in constructing smooth piecewise surfaces, people usually consider only geometric continuity, namely, $\mathrm{G}^{n}$ continuity, which is irrelevant to the selected parameters. In practical application, adjacent surfaces usually only need to reach G^1^ continuity, which means that adjacent surfaces need to possess a common tangent plane or surface normal at any point on their common boundary; while in some situations with high demand for smoothness, adjacent surfaces are required to reach G^2^ continuity (namely, curvature continuity) [3]. At present, owing to their simple and intuitive definition and some outstanding properties, Bézier parametric surfaces have long been one of the important methods for representing surfaces in the CAD/CAM system. However, the Bézier model still has a weakness that the shape of a Bézier surface is uniquely determined by its control mesh points. In order to overcome this weakness, scholars proposed rational Bézier surfaces and NURBS surfaces, whose shapes can be modified or adjusted by changing their weight factors on the condition of given control mesh points. However, the introduction of rational fractions also brought in some other drawbacks such as complex calculation, inconvenience for integrals, higher-order expressions resulting from repeated differentiation, etc. [4]. In addition, though the smooth continuity technologies of Bézier, rational Bézier and NURBS surfaces, which can be used to construct various complex surfaces, have been widely researched in [5–10], the drawbacks of these surfaces also exist in the piecewise surfaces composed of them. All of these might get the design of complex surfaces in trouble (such as the problem of shape adjustment).

In order to reserve the advantages of Bézier model and improve the shape adjustability of curves and surfaces, scholars have constructed many Bézier curves and surfaces with shape parameters [11–18]. The common features of these curves and surfaces are as follows: (1) they inherit most of properties of Bézier curves and surfaces; (2) they all have shape parameters used to adjust the shape of these curves and surfaces handily; (3) the absence of rational fractions in their expressions makes them simpler than rational Bézier and NURBS curves and surfaces. Thus these curves and surfaces have extensive applications in describing complex curves and surfaces. However, the expressions of these Bézier curves and surfaces with shape parameters are polynomials; and consequently, they face the problem of smooth continuity in constructing complex curves and surfaces. Therefore, when researchers defined their curves and surfaces with shape parameters in [11–15], they also further studied the C^1^, C^2^ or G^1^, G^2^ continuity conditions of their proposed curves, but the continuity conditions of these surfaces have not been studied until now (note: the continuity conditions of the surfaces in [16–18] are also not studied). Compared with the research on smooth continuity between Bézier curves with shape parameters, the corresponding research on Bézier surfaces with shape parameters has not been extensively done and the relevant research results are relatively few. In this paper, we make some improvements to the Bézier-like surfaces in [17] and construct a kind of high-order generalized Bézier-like surfaces associated with multiple shape parameters. To improve the ability of describing complex surfaces by using the proposed surfaces, we lay emphasis on the study of G^2^ continuity conditions of these surfaces.

The remainder of the paper is organized as follows. The definition of generalized Bézier-like surfaces is given in Section 2. In Section 3, we propose the G^2^ continuity conditions for generalized Bézier-like surfaces. Some examples of G^2^ smooth continuity between generalized Bézier-like surfaces are given in Section 4. In Section 5, we discuss the shape adjustment of piecewise surfaces. At last, some conclusions are given in Section 6.

## Generalized Bézier-like surfaces with shape parameters

### Definition of Bernstein-like basis functions

#### Definition 1

For any $t \in [0, 1]$, the following polynomial functions of *t*

1 $$ b_{i,n}(t;\lambda ) = \biggl(1 + \frac{3C_{n - 2}^{i - 1} + C_{n - 1}^{i} - C_{n}^{i}}{C_{n}^{i}}\lambda - \frac{2C_{n - 1}^{i}}{C_{n}^{i}}\lambda t + \lambda t^{2}\biggr)C_{n}^{i}t^{i}(1 - t)^{n - i}\quad (i = 0,1, \ldots,n ) $$

are called the Bernstein-like basis functions of degree *n* [17], where $n \ge2$ $C_{n}^{i} = \frac{n !}{i !(n - i) !}$, $C_{n - 2}^{ - 1} = C_{n - 1}^{n} = C_{n - 2}^{n - 1} = 0$; $\lambda \in [ - 1, 1]$ is a shape parameter.

**Figure 1:**
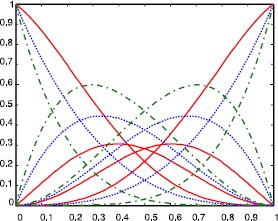
**The cubic Bernstein-like basis functions with the shape parameter** $\pmb{\lambda = -1, 0\mbox{ and }1}$ **.**

It can be easily proved that the Bernstein-like basis functions $b_{i,n}(t;\lambda )$ share many properties with classical Bernstein basis functions, such as non-negativity, property of weight distribution, symmetry, linear independence, etc. Especially when the shape parameter $\lambda = 0$, $b_{i,n}(t;\lambda )$ ($i = 0,1, \ldots,n$) degrade into classical Bernstein basis functions of degree *n*. Figure 1 shows the graphs of the cubic Bernstein-like basis functions with the shape parameter *λ* taking different values, where the red solid lines, blue dotted lines and green dot dash lines are the graphs of the basis functions with the shape parameter $\lambda= -1, 0\mbox{ and }1$, respectively.

By the Bernstein-like basis functions in (1), a Bézier-like curve of degree *n* associated with the shape parameter *λ* can be defined as follows [17]:

2 $$ \boldsymbol{C}(t;\lambda ) = \sum_{i = 0}^{n} \boldsymbol{P}_{i}b_{i,n}(t;\lambda ),\quad t \in [0, 1], $$

where $\boldsymbol{P}_{i}\in \mathrm{R}^{d}$ ($d = 2,3$; $i = 0,1, \ldots,n$; $n \ge 2$) are control points of the curve; $b_{i,n}(t;\lambda )$ ($i = 0,1, \ldots,n$) are Bernstein-like basis functions of degree *n* defined by (1).

### Construction of generalized Bézier-like surfaces

Similar to the form of classical tensor-product Bézier surfaces (but slightly different), a kind of generalized Bézier-like surfaces associated with $m+2$ shape parameters can be constructed by blending $m+1$ Bézier-like curves with independent shape parameters.

#### Definition 2

Given $m \times n$ control mesh points $\boldsymbol{P}_{i,j}\in \mathrm{R}^{3}$ ($i = 0,1, \ldots,m$; $j = 0,1, \ldots,n$; $m,n \ge 2$) in 3D space, *λ* and $\gamma_{i}$ ($i = 0,1, \ldots,m$) are constants and $- 1 < \lambda,\gamma_{i} \le 1$, the generalized Bézier-like surfaces of degree $(m, n)$ can be defined as

3 $$ \boldsymbol{S}(u,v;\lambda,\gamma_{i}) = \sum _{i = 0}^{m} \Biggl[ b_{i,m}(u; \lambda )\sum _{j = 0}^{n} b_{j,n}(v; \gamma_{i})\boldsymbol{P}_{i,j} \Biggr]\quad (0 \le u, v \le 1), $$

where $b_{i,m}(u;\lambda )$ and $b_{j,n}(v;\gamma_{i})$ ($i = 0,1, \ldots,m$; $j = 0,1, \ldots,n$) are Bernstein-like basis functions of degree *m* and *n* defined by (1), respectively, *m* and *n* are positive integers, and $m,n \ge 2$; *λ*, $\gamma_{i}$ ($i = 0,1, \ldots,m$) are shape control parameters of the surfaces.

#### Remark 1

The generalized Bézier-like surfaces inherited most of the properties of classical Bézier surface, such as angular point interpolation property, boundary property, degeneracy, symmetry, convex hull property, geometric, affine invariance, etc.

#### Remark 2

The generalized Bézier-like surfaces have the following advantages: on the condition of keeping the control mesh points of a surface unchanged, the shape of the surface can also be modified flexibly by changing its shape parameters, and the surface has $3^{m+2}-1$ ways to approximate its control mesh. Especially when all the shape parameters equal 0, the generalized Bézier-like surfaces degenerate into classical Bézier surfaces of degree $(m, n)$.

### Influence rule of the shape parameters on generalized Bézier-like surfaces

In order to adjust the shape of the generalized Bézier-like surfaces effectively, the influence rule of the shape parameters on them is analyzed in details in this section. In other words, how will the shape of the surfaces change when one or multiple parameters change is particularly demonstrated to enable designers to modify the shape of the surfaces purposefully and efficiently.

#### Proposition 1

*On the condition of keeping the control mesh points and the shape parameters* $\gamma_{i}$ ($i = 0,1, \ldots,m$) *of the generalized Bézier*-*like surfaces unchanged*,

- *the generalized Bézier*-*like surfaces will get nearer to* (*or farther away from*) *their control mesh when the shape parameter* *λ* *increases* (*or decreases*).
- *changing the value of the shape parameter* *λ*, *the position and shape of the boundary curves* $\boldsymbol{S}(0,v;\lambda,\gamma_{i})$ *and* $\boldsymbol{S}(1,v;\lambda,\gamma_{i})$ *as well as the position of the four corners of the generated surfaces will keep unchanged*, *while the position and shape of the boundary curves* $\boldsymbol{S}(u,0;\lambda,\gamma_{i})$ *and* $\boldsymbol{S}(u,1;\lambda,\gamma_{i})$ *will change*.

**Figure 2:**
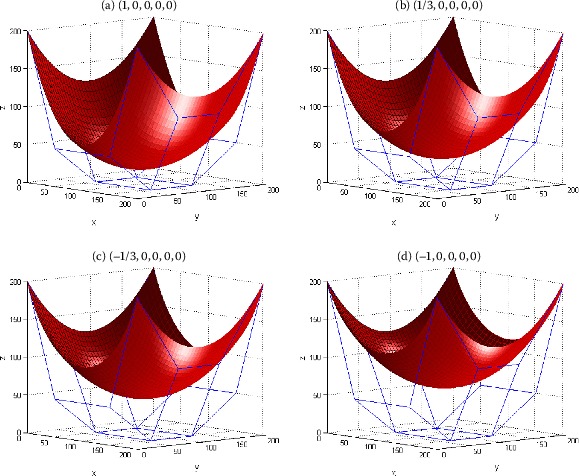
**Generalized Bézier-like surfaces of degree** $\pmb{(3, 3)}$ **with different shape parameter** ***λ*** **.**

Figure 2 gives an example to show the shape adjustment of the generalized Bézier-like surfaces by using the shape parameter *λ*. The surface in Figure 2 is a generalized Bézier-like one of degree $(3, 3)$, whose shape is adjusted by changing the value of the shape parameter *λ* on the condition of the control mesh points and the remaining shape parameters $\gamma_{i}$ ($i = 0,1, \ldots,m$) keeping unchanged. In order to facilitate the discussion, the shape parameters of the generalized Bézier-like surfaces can be written in vector form as $(\lambda,\gamma_{0},\gamma_{1},\lambda_{2},\gamma_{3})$. It can be seen from Figure 2 that the shape change of the generalized Bézier-like surfaces accords with the conclusion of Proposition 1.

#### Proposition 2

*On the condition of keeping the control mesh points and the shape parameter* *λ* *of the generalized Bézier*-*like surfaces unchanged*,

- *with the increase* (*or decrease*) *of the shape parameters* $\gamma_{i}$ ($i = 0,1, \ldots,m$), *the generalized Bézier*-*like surfaces will gradually get nearer to* (*or farther away from*) *their control mesh along the control polygon composed of the points* $\boldsymbol{P}_{i, j}$ ($j = 0,1, \ldots,n$). *Therefore the shape parameters* $\gamma_{i}$ ($i = 0,1, \ldots,m$) *mainly control the shape of the generalized Bézier*-*like surfaces near the control points* $\boldsymbol{P}_{i, 0},\boldsymbol{P}_{i, 1}, \ldots,\boldsymbol{P}_{i, n}$.
- *with the single change of the shape parameter* $\gamma_{0}$ (*or* $\gamma_{m}$), *the position and shape of the boundary curve* $\boldsymbol{S}(0,v;\lambda,\gamma_{i})$ (*or* $\boldsymbol{S}(1,v;\lambda,\gamma_{i})$) *of generalized Bézier*-*like surfaces will change*, *while the position and shape of the other three boundary curves as well as the position of the four corners of the surfaces remain unchanged*. *With the change of the shape parameters* $\gamma_{i}$ ($i = 1,2, \ldots,m - 1$), *the position and shape of the four boundary curves as well as the position of the four corners of the generated surfaces remain unchanged*.

**Figure 3:**
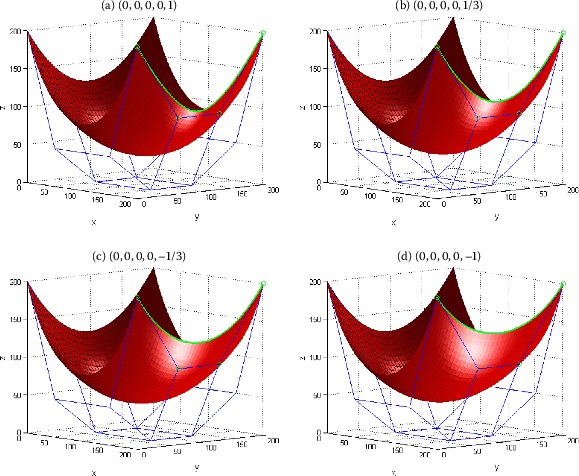
**Generalized Bézier-like surfaces of degree** $\pmb{(3, 3)}$ **with different shape parameter** $\pmb{\gamma_{3}}$ **.**

Figure 3 gives an example to show the shape adjustment of the generalized Bézier-like surfaces by using the shape parameter $\gamma_{3}$. The surface in Figure 3 is a generalized Bézier-like one of degree $(3, 3)$, whose control mesh points are the same as those in Figure 2. With the control mesh points and the values of the shape parameters *λ*, $\gamma_{i}$ ($i = 0,1,2$) kept unchanged, the local shape of the generalized Bézier-like surface is adjusted by changing the shape parameter $\gamma_{3}$. It can be seen from Figure 3 that the shape of the surface changes apparently near the control points $\boldsymbol{P}_{3, 0}$, $\boldsymbol{P}_{3, 1}$, $\boldsymbol{P}_{3, 2}$, $\boldsymbol{P}_{3, 3}$, which are marked as circles. What is more, the position and shape of the boundary curve $\boldsymbol{S}(1,v;\lambda,\gamma_{i})$ (which is marked in blue) changes with the value change of $\gamma_{3}$, while the position and shape of the other three boundary curves remain unchanged. The influence rules of the shape parameters $\gamma_{i}$ ($i = 0,1,2$) on generalized Bézier-like surfaces can be discussed similarly.

#### Remark 3

On the basis of the conclusion of Proposition 1 and Proposition 2 and the control mesh points kept unchanged, we have the following:

- with the simultaneous increase (or decrease) of the shape parameters *λ*, $\gamma_{i}$ ($i = 0,1, \ldots,m$), the generalized Bézier-like surfaces gradually get nearer to (or farther away from) their control mesh.
- fixing the values of the shape parameters *λ*, $\gamma_{0}$, $\gamma_{m}$, the shape of the generalized Bézier-like surfaces can be adjusted by changing the shape parameters $\gamma_{i}$ ($i = 1,2, \ldots,m - 1$) with the four boundary curves of the surfaces remaining unchanged.

**Figure 4:**
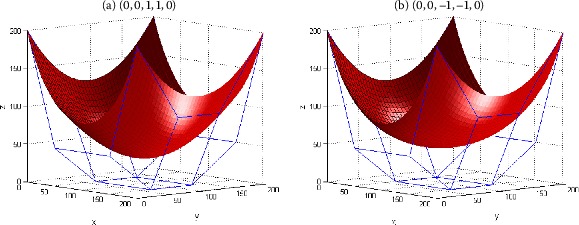
**Shape modification of generalized Bézier-like surfaces with fixed boundary.**

Figure 4 gives an example to show the shape adjustment of the generalized Bézier-like surfaces by using the shape parameters $\gamma_{i}$ ($i = 1,2$). It can be seen from Figure 4 that, on the condition of keeping the four boundary curves of the surface in Figure 4 unchanged, the concavo-convex shape of the surface can be flexibly modified by changing the shape parameters $\gamma_{1}$, $\gamma_{2}$. To sum up, with given control mesh, designers can conveniently adjust both the local and global shape of generalized Bézier-like surfaces by changing the shape parameters in practical application.

## G^2^ continuity conditions for generalized Bézier-like surfaces

In designing complex surfaces, designers usually need to make adjacent surfaces reach G^1^ continuity, and G^2^ continuity is required in some situations with high demand for smoothness. If two adjacent surfaces need to reach G^2^ continuity, they must reach G^1^ continuity first. In other words, the G^2^ geometric continuity conditions contain the G^1^ ones, so the G^2^ continuity conditions of the surfaces are only covered in this section. In order to facilitate our discussion, suppose that there are the following two generalized Bézier-like surfaces needing to reach G^2^ smooth continuity:

4 $$ \textstyle\begin{cases} \boldsymbol{S}_{1}(u,v;\lambda_{1},\gamma_{i,1}) = \sum_{i = 0}^{m_{1}} [ b_{i, m_{1}}(u; \lambda_{1})\sum_{j = 0}^{n_{1}} b_{j,n_{1}}(v; \gamma_{i, 1})\boldsymbol{P}_{i,j}^{1} ], \\ \boldsymbol{S}_{2}(u,v;\lambda_{2},\gamma_{i,2}) = \sum_{i = 0}^{m_{2}} [ b_{i, m_{2}}(u; \lambda_{2})\sum_{j = 0}^{n_{2}} b_{j,n_{2}}(v; \gamma_{i,2})\boldsymbol{P}_{i,j}^{2} ], \end{cases} $$

where $- 1 \le \lambda_{1},\lambda_{2},\gamma_{i,1},\gamma_{i,2} \le 1$, $\boldsymbol{P}_{i,j}^{1}$ ($i = 0,1, \ldots,m_{1}$; $j = 0,1, \ldots,n_{1}$) and $\boldsymbol{P}_{i,j}^{2}$ ($i = 0,1, \ldots,m_{2}$; $j = 0,1, \ldots,n_{2}$) are control mesh points of the surfaces $\boldsymbol{S}_{1}(u,v;\lambda_{1},\gamma_{i,1})$ and $\boldsymbol{S}_{2}(u,v;\lambda_{2},\gamma_{i,2})$, respectively. Because surfaces have their directivity, so there are three ways for the proposed surfaces to reach G^2^ smooth continuity at the joint: smooth continuity in the *u* direction, smooth continuity in the direction of *u* and *v*, and smooth continuity in the *v* direction.

### Smooth continuity in the *u* direction

#### Theorem 1

*If the two adjacent generalized Bézier*-*like surfaces* $\boldsymbol{S}_{1}(u,v;\lambda_{1},\gamma_{i,1})$ *and* $\boldsymbol{S}_{2}(u,v; \lambda_{2},\gamma_{i,2})$ *satisfy all the following conditions*:

5 $$ \textstyle\begin{cases} m_{1} = m_{2},\qquad \lambda_{1} = \lambda_{2}, \\ \boldsymbol{P}_{i,n_{1}}^{1} = \boldsymbol{P}_{i,0}^{2}\quad (i = 0,1, \ldots,m_{1}), \\ \frac{\boldsymbol{P}_{i,n_{1}}^{1} - \boldsymbol{P}_{i,n_{1} - 1}^{1}}{n_{2} + 2\gamma_{i,2}} = f\frac{\boldsymbol{P}_{i,1}^{2} - \boldsymbol{P}_{i,0}^{2}}{n_{1} + 2\gamma_{i,1}}\quad (i = 0,1, \ldots m_{1}), \\ (2\gamma_{i,1} + 4\gamma_{i,1}n_{1} - n_{1} + n_{1}^{2})(\boldsymbol{P}_{i,n_{ 1}}^{1} - \boldsymbol{P}_{i,n_{ 1} - 1}^{1})\\ \qquad {} + (10\gamma_{i,1} - 4\gamma_{i,1}n_{1} + n_{1} - n_{1}^{2})(\boldsymbol{P}_{i,n_{ 1} - 1}^{1} - \boldsymbol{P}_{i,n_{ 1} - 2}^{1}) \\ \quad = f^{2} [ (2\gamma_{i,2} + 4\gamma_{i,2}n_{2} - n_{2} + n_{2}^{2})(\boldsymbol{P}_{i,0}^{2} - \boldsymbol{P}_{i,1}^{2})\\ \qquad {} + (10\gamma_{i,2} - 4\gamma_{i,2}n_{2} + n_{2} - n_{2}^{2})(\boldsymbol{P}_{i,1}^{2} - \boldsymbol{P}_{i,2}^{2}) ] \\ \quad (i = 0,1, \ldots,m_{1}) \end{cases} $$

*surfaces* $\boldsymbol{S}_{1}$ *and* $\boldsymbol{S}_{2}$ *reach G* ^2^ *smooth continuity in the* *u* *direction at the joint*, *where* $f > 0$ *is a real constant*.

#### Proof

If $\boldsymbol{S}_{1}(u,v;\lambda_{1},\gamma_{i,1})$ and $\boldsymbol{S}_{2}(u,v;\lambda_{2},\gamma_{i,2})$ need to reach G^2^ smooth continuity in the *u* direction at the joint, they are required to reach G^1^ smooth continuity at the joint first. In other words, the two surfaces need to possess a common tangent plane or surface normal at any point on their common boundary [1–3, 19, 20].

The same is true of G^1^ smooth continuity. That is, the two surfaces are required to reach G^0^ continuity in the *u* direction first (namely, they need to possess a common boundary), which means

$$\boldsymbol{S}_{1}(u,1;\lambda_{1},\gamma_{i,1}) = \boldsymbol{S}_{2}(u,0;\lambda_{2},\gamma_{i,2}). $$

According to boundary properties of the surfaces in (3), the equation above can be expressed as

6 $$ \sum_{i = 0}^{m_{1}} b_{i,m_{1}}(u; \lambda_{1})\boldsymbol{P}_{i, n_{1}}^{1} = \sum _{i = 0}^{m_{2}} b_{i,m_{2}}(u; \lambda_{2})\boldsymbol{P}_{i, 0}^{2}. $$

Based on the linear independence of the Bernstein-like basis functions in (1), (6) can be simplified by coefficients comparing as follows:

7 $$ \textstyle\begin{cases} m_{1} = m_{2}, \\ \boldsymbol{P}_{i,n_{1}}^{1} = \boldsymbol{P}_{i,0}^{2}\quad (i = 0,1, \ldots,m_{1}), \\ \lambda_{1} = \lambda_{2}. \end{cases} $$

According to the definition of G^1^ smooth continuity, the two generalized Bézier-like surfaces are required to possess a common tangent plane at any point on their common boundary (namely, their tangential derivatives across the boundary should be continuous) [3, 19, 20], thus they need to satisfy

8 $$ \begin{aligned}[b] &\frac{\partial}{\partial v}\boldsymbol{S}_{1}(u,1; \lambda_{1},\gamma_{i,1}) \times \frac{\partial}{ \partial u} \boldsymbol{S}_{1}(u,1;\lambda_{1},\gamma_{i,1}) \\ &\quad =f(u)\frac{\partial}{\partial v}\boldsymbol{S}_{2}(u,0; \lambda_{2},\gamma_{i,2}) \times \frac{\partial}{ \partial u} \boldsymbol{S}_{2}(u,0;\lambda_{2},\gamma_{i,2}), \end{aligned} $$

where $f(u)$ is the scaling factor between their normal vectors at the joint and $f(u) > 0$. For simplifying the calculation process, the Faux method [3, 19, 20] is usually adopted in practical applications, by which (8) can be simplified as

9 $$ \frac{\partial}{\partial v}\boldsymbol{S}_{1}(u,1;\lambda_{1}, \gamma_{i,1}) = f \frac{\partial}{ \partial v}\boldsymbol{S}_{2}(u,0; \lambda_{2},\gamma_{i,2}), $$

where $f > 0$ is a real constant, (9) means that their cross-border tangent vector across their common boundary should be continuous.

By calculating the cross-border tangent vector of $\boldsymbol{S}_{1}(u,v;\lambda_{1},\gamma_{i,1})$ and $\boldsymbol{S}_{2}(u,v;\lambda_{2},\gamma_{i,2})$ in the *v* direction, and substituting them into (9), we have

10 $$ \sum_{i = 0}^{m_{1}} (n_{1} + 2 \gamma_{i,1})b_{i,m_{1}}(u;\lambda_{1}) \bigl( \boldsymbol{P}_{i,n_{1}}^{1} - \boldsymbol{P}_{i,n_{1} - 1}^{1} \bigr) = f\sum_{i = 0}^{m_{2}} (n_{2} + 2\gamma_{i,2})b_{i,m_{2}}(u;\lambda_{2}) \bigl( \boldsymbol{P}_{i,1}^{2} - \boldsymbol{P}_{i,0}^{2} \bigr). $$

Combining with the results in (7), (10) can be simplified as

11 $$ \frac{\boldsymbol{P}_{i,n_{1}}^{1} - \boldsymbol{P}_{i,n_{1} - 1}^{1}}{n_{2} + 2\gamma_{i,2}} = f\frac{\boldsymbol{P}_{i,1}^{2} - \boldsymbol{P}_{i,0}^{2}}{n_{1} + 2\gamma_{i,1}}\quad (i = 0,1, \ldots,m_{1}). $$

In addition, under the condition of G^1^ smooth continuity, the two surfaces need to possess the same normal curvature at any point on their common boundary [3, 19], so the two surfaces also need to satisfy

12 $$ \begin{aligned}[b] \frac{\partial^{2}}{\partial v^{2}}\boldsymbol{S}_{1}(u,1; \lambda_{1},\gamma_{i,1}) &= f^{2}\frac{\partial^{2}}{\partial v^{2}} \boldsymbol{S}_{2}(u,0;\lambda_{2},\gamma_{i,2}) + 2f g(u)\frac{\partial^{2}}{\partial u\,\partial v}\boldsymbol{S}_{2}(u,0;\lambda_{2}, \gamma_{i,2}) \\ &\quad{} + g^{2}(u)\frac{\partial^{2}}{\partial u\,\partial u}\boldsymbol{S}_{2}(u,0; \lambda_{2},\gamma_{i,2}) + c\frac{\partial}{ \partial v} \boldsymbol{S}_{2}(u,0;\lambda_{2},\gamma_{i,2})\\ &\quad {} + d(u)\frac{\partial}{\partial u}\boldsymbol{S}_{2}(u,0;\lambda_{2}, \gamma_{i,2}), \end{aligned} $$

where $g(u)$ and $d(u)$ are linear functions of *u*, *c* is an arbitrary constant and *f* is the same as (9). To facilitate the operation and calculation in practical applications, usually set $g(u) = d(u) = c = 0$, so (12) can be further simplified as

13 $$ \frac{\partial^{2}}{\partial v^{2}}\boldsymbol{S}_{1}(u,1;\lambda_{1}, \gamma_{i,1}) = f^{2}\frac{\partial^{2}}{\partial v^{2}}\boldsymbol{S}_{2}(u,0; \lambda_{2},\gamma_{i,2}). $$

According to (1), the second-order derivatives of the Bernstein-like basis functions $b_{i,n}(t; \lambda )$ ($i = 0,1, \ldots,n$; $n \ge 2$) at terminal points are

14 $$\begin{aligned}& b''_{i,n}(0;\lambda ) = \textstyle\begin{cases} 2\lambda + (4\lambda - 1)n + n^{2}, &i = 0, \\ 8\lambda + (2 - 8\lambda )n - 2n^{2},& i = 1, \\ - 10\lambda + (4\lambda - 1)n + n^{2}, &i = 2 , \\ 0,& i = 3,4, \ldots,n , \end{cases}\displaystyle \end{aligned}$$

15 $$\begin{aligned}& b''_{i,n}(1;\lambda ) = \textstyle\begin{cases} - 10\lambda + (4\lambda - 1)n + n^{2}, &i = n - 2, \\ 8\lambda + (2 - 8\lambda )n - 2n^{2}, &i = n - 1, \\ 2\lambda + (4\lambda - 1)n + n^{2}, &i = n, \\ 0,& i = 1,2, \ldots,n - 3. \end{cases}\displaystyle \end{aligned}$$

Thus, on the basis of (4), (14) and (15), we have

16 $$ \textstyle\begin{cases} \frac{\partial^{2}}{\partial v^{2}}\boldsymbol{S}_{1}(u,1;\lambda_{1},\gamma_{i,1}) = \sum_{i = 0}^{m_{1}} [ b_{i,m_{1}}(u; \lambda_{1})\sum_{j = 0}^{n_{1}} b''_{j,n_{1}}(1; \gamma_{i,1})\boldsymbol{P}_{i,j}^{1} ] \\ \hphantom{ \frac{\partial^{2}}{\partial v^{2}}\boldsymbol{S}_{1}(u,1;\lambda_{1},\gamma_{i,1})} = \sum_{i = 0}^{m_{1}} b_{i,m_{1}}(u; \lambda_{1}) [ (2\gamma_{i,1} + 4\gamma_{i,1}n_{1} - n_{1} + n_{1}^{2})(\boldsymbol{P}_{i,n_{ 1}}^{1} - \boldsymbol{P}_{i,n_{ 1} - 1}^{1})\\ \hphantom{ \frac{\partial^{2}}{\partial v^{2}}\boldsymbol{S}_{1}(u,1;\lambda_{1},\gamma_{i,1}) =} {} + (10\gamma_{i,1} - 4\gamma_{i,1}n_{1} + n_{1} - n_{1}^{2})(\boldsymbol{P}_{i,n_{ 1} - 1}^{1} - \boldsymbol{P}_{i,n_{ 1} - 2}^{1}) ], \\ \frac{\partial^{2}}{\partial v^{2}}\boldsymbol{S}_{2}(u,0;\lambda_{2},\gamma_{i,2}) = \sum_{i = 0}^{m_{2}} [ b_{i,m_{2}}(u; \lambda_{2})\sum_{j = 0}^{n_{2}} b''_{j,n_{2}}(0; \gamma_{i,2})\boldsymbol{P}_{i,j}^{2} ] \\ \hphantom{\frac{\partial^{2}}{\partial v^{2}}\boldsymbol{S}_{2}(u,0;\lambda_{2},\gamma_{i,2})}= \sum_{i = 0}^{m_{2}} b_{i,m_{2}}(u; \lambda_{2}) [ (2\gamma_{i,2} + 4\gamma_{i,2}n_{2} - n_{2} + n_{2}^{2})(\boldsymbol{P}_{i,0}^{2} - \boldsymbol{P}_{i,1}^{2})\\ \hphantom{\frac{\partial^{2}}{\partial v^{2}}\boldsymbol{S}_{2}(u,0;\lambda_{2},\gamma_{i,2})=}{} + (10\gamma_{i,2} - 4\gamma_{i,2}n_{2} + n_{2} - n_{2}^{2})(\boldsymbol{P}_{i,1}^{2} - \boldsymbol{P}_{i,2}^{2}) ]. \end{cases} $$

Substituting the second-order cross-border tangent vector above into (13), we can get

17 $$ \begin{aligned}[b] &\sum_{i = 0}^{m_{1}} b_{i,m_{1}}(u; \lambda_{1}) \bigl[ \bigl(2\gamma_{i,1} + 4\gamma_{i,1}n_{1} - n_{1} + n_{1}^{2} \bigr) \bigl(\boldsymbol{P}_{i,n_{ 1}}^{1} - \boldsymbol{P}_{i,n_{ 1} - 1}^{1} \bigr)\\ &\qquad {} + \bigl(10\gamma_{i,1} - 4\gamma_{i,1}n_{1} + n_{1} - n_{1}^{2}\bigr) \bigl( \boldsymbol{P}_{i,n_{ 1} - 1}^{1} - \boldsymbol{P}_{i,n_{ 1} - 2}^{1} \bigr) \bigr] \\ &\quad = f^{2}\sum_{i = 0}^{m_{2}} b_{i,m_{2}}(u; \lambda_{2}) \bigl[ \bigl(2\gamma_{i,2} + 4\gamma_{i,2}n_{2} - n_{2} + n_{2}^{2} \bigr) \bigl(\boldsymbol{P}_{i,0}^{2} - \boldsymbol{P}_{i,1}^{2} \bigr)\\ &\qquad {} + \bigl(10\gamma_{i,2} - 4\gamma_{i,2}n_{2} + n_{2} - n_{2}^{2}\bigr) \bigl(\boldsymbol{P}_{i,1}^{2} - \boldsymbol{P}_{i,2}^{2}\bigr) \bigr]. \end{aligned} $$

Finally, combining with the conclusions of (7) and (11), (17) can be written as

18 $$ \begin{aligned}[b] & \bigl(2\gamma_{i,1} + 4\gamma_{i,1}n_{1} - n_{1} + n_{1}^{2}\bigr) \bigl( \boldsymbol{P}_{i,n_{ 1}}^{1} - \boldsymbol{P}_{i,n_{ 1} - 1}^{1} \bigr) + \bigl(10\gamma_{i,1} - 4\gamma_{i,1}n_{1} + n_{1} - n_{1}^{2}\bigr) \bigl( \boldsymbol{P}_{i,n_{ 1} - 1}^{1} - \boldsymbol{P}_{i,n_{ 1} - 2}^{1} \bigr)\hspace{-20pt} \\ &\quad = f^{2} \bigl[ \bigl(2\gamma_{i,2} + 4 \gamma_{i,2}n_{2} - n_{2} + n_{2}^{2} \bigr) \bigl(\boldsymbol{P}_{i,0}^{2} - \boldsymbol{P}_{i,1}^{2} \bigr) + \bigl(10\gamma_{i,2} - 4\gamma_{i,2}n_{2} + n_{2} - n_{2}^{2}\bigr) \bigl(\boldsymbol{P}_{i,1}^{2} - \boldsymbol{P}_{i,2}^{2}\bigr) \bigr]\hspace{-20pt} \\ &\quad (i = 0,1, \ldots,m_{1}). \end{aligned} $$

To sum up, if the two surfaces $\boldsymbol{S}_{1}(u,v;\lambda_{1},\gamma_{i,1})$ and $\boldsymbol{S}_{2}(u,v;\lambda_{2},\gamma_{i,2})$ satisfy (7), (11) and (18) simultaneously, they reach G^2^ smooth continuity in the *u* direction at the joint, and Theorem 1 gets proved. Obviously, if the two surfaces satisfy both (7) and (11), they reach G^1^ smooth continuity in the *u* direction at the joint. □

### Smooth continuity in the direction of *u* and *v*

#### Theorem 2

*If the two adjacent generalized Bézier*-*like surfaces* $\boldsymbol{S}_{1}(u,v;\lambda_{1},\gamma_{i,1})$ *and* $\boldsymbol{S}_{2}(u,v; \lambda_{2},\gamma_{i,2})$ *satisfy all the following conditions*:

19 $$ \textstyle\begin{cases} m_{1} = n_{2},\qquad \lambda_{1} = \gamma_{0,2} = \gamma_{1,2} = \gamma_{2,2}, \\ \boldsymbol{P}_{i,n_{1}}^{1} = \boldsymbol{P}_{0,j}^{2}\quad (i = j = 0,1,\ldots,m_{1}), \\ \frac{\boldsymbol{P}_{i,n_{1}}^{1} - \boldsymbol{P}_{i,n_{1} - 1}^{1}}{m_{2} + 2\lambda_{2}} = f\frac{\boldsymbol{P}_{1,j}^{2} - \boldsymbol{P}_{0,j}^{2}}{n_{1} + 2\gamma_{i,1}}\quad (i = j = 0,1, \ldots, m_{1}), \\ (2\gamma_{i,1} + 4\gamma_{i,1}n_{1} - n_{1} + n_{1}^{2})(\boldsymbol{P}_{i,n_{ 1}}^{1} - \boldsymbol{P}_{i,n_{ 1} - 1}^{1})\\ \qquad {} + (10\gamma_{i,1} - 4\gamma_{i,1}n_{1} + n_{1} - n_{1}^{2})(\boldsymbol{P}_{i,n_{ 1} - 1}^{1} - \boldsymbol{P}_{i,n_{ 1} - 2}^{1}) \\ \quad = f^{2} [ (2\lambda_{2} + 4\lambda_{2}m_{2} - m_{2} + m_{2}^{2})(\boldsymbol{P}_{0,j}^{2} - \boldsymbol{P}_{1,j}^{2})\\ \qquad {} + (10\lambda_{2} - 4\lambda_{2}m_{2} + m_{2} - m_{2}^{2})(\boldsymbol{P}_{1,j}^{2} - \boldsymbol{P}_{2,j}^{2}) ] \\ \quad (i = j = 0,1, \ldots,m_{1}) \end{cases} $$

*surfaces* $\boldsymbol{S}_{1}$ *and* $\boldsymbol{S}_{2}$ *reach G* ^2^ *smooth continuity in the direction of* *u* *and* *v*.

#### Proof

Suppose that the surfaces $\boldsymbol{S}_{1}(u,v;\lambda_{1},\gamma_{i,1})$ and $\boldsymbol{S}_{2}(u,v;\lambda_{2},\gamma_{i,2})$ need to reach G^2^ smooth continuity in the *u* direction of $\boldsymbol{S}_{1}(u,v;\lambda_{1},\gamma_{i,1})$ and the *v* direction of $\boldsymbol{S}_{2}(u,v;\lambda_{2},\gamma_{i,2})$. Similar to the derivation in Theorem 1, the two surfaces need to satisfy G^1^ continuity conditions in the direction of *u* and *v* first, which means

20 $$ \textstyle\begin{cases} m_{1} = n_{2}, \\ \boldsymbol{P}_{i,n_{1}}^{1} = \boldsymbol{P}_{0,j}^{2}\quad (i = j = 0,1,\ldots,m_{1}), \\ \lambda_{1} = \gamma_{0,2} = \gamma_{1,2}, \\ \frac{\boldsymbol{P}_{i,n_{1}}^{1} - \boldsymbol{P}_{i,n_{1} - 1}^{1}}{m_{2} + 2\lambda_{2}} = f\frac{\boldsymbol{P}_{1,j}^{2} - \boldsymbol{P}_{0,j}^{2}}{n_{1} + 2\gamma_{i,1}}\quad (i = j = 0,1, \ldots,m_{1}), \end{cases} $$

where $f > 0$ is a real constant.

Furthermore, under the condition of G^1^ smooth continuity, the two surfaces also need to possess the same normal curvature at any point on their common boundary [3, 19], so they also need to satisfy

21 $$ \frac{\partial^{2}}{\partial v^{2}}\boldsymbol{S}_{1}(u,1;\lambda_{1}, \gamma_{i,1}) = f^{2}\frac{\partial^{2}}{\partial u^{2}}\boldsymbol{S}_{2}(0,v; \lambda_{2},\gamma_{i,2}), $$

where *f* is the same as (20).

Finally, by calculating the second-order cross-border tangent vector in (21) by the method in Theorem 1 and substituting it into (21), we have

22 $$\begin{aligned} &\sum_{i = 0}^{m_{1}} b_{i,m_{1}}(u; \lambda_{1}) \bigl[ \bigl(2\gamma_{i,1} + 4\gamma_{i,1}n_{1} - n_{1} + n_{1}^{2} \bigr) \bigl(\boldsymbol{P}_{i,n_{ 1}}^{1} - \boldsymbol{P}_{i,n_{ 1} - 1}^{1} \bigr) \\ &\qquad {} + \bigl(10\gamma_{i,1} - 4\gamma_{i,1}n_{1} + n_{1} - n_{1}^{2}\bigr) \bigl( \boldsymbol{P}_{i,n_{ 1} - 1}^{1} - \boldsymbol{P}_{i,n_{ 1} - 2}^{1} \bigr) \bigr] \\ &\begin{aligned}[b]&\quad = f^{2}\Biggl[ \bigl(2\lambda_{2} + 4 \lambda_{2}m_{2} - m_{2} + m_{2}^{2} \bigr)\sum_{j = 0}^{n_{ 2}} b_{j,n_{2}}(v; \gamma_{0,2})\boldsymbol{P}_{0,j}^{2}\\ &\qquad {} + \bigl(8 \lambda_{2} - 8\lambda_{2}m_{2} + 2m_{2} - 2m_{2}^{2}\bigr)\sum_{j = 0}^{n_{ 2}} b_{j,n_{2}}(v; \gamma_{1,2})\boldsymbol{P}_{1,j}^{2} \\ &\qquad {} + \bigl( - 10\lambda_{2} + 4\lambda_{2}m_{2} - m_{2} + m_{2}^{2}\bigr)\sum _{j = 0}^{n_{ 2}} b_{j,n_{2}}(v; \gamma_{2,2}) \boldsymbol{P}_{2,j}^{2} \Biggr]. \end{aligned} \end{aligned}$$

Obviously, when $\gamma_{0,2} = \gamma_{1,2} = \gamma_{2,2}$, combining the results in (20), (22) can be simplified as

23 $$ \begin{aligned}[b] &\bigl(2\gamma_{i,1} + 4\gamma_{i,1}n_{1} - n_{1} + n_{1}^{2}\bigr) \bigl( \boldsymbol{P}_{i,n_{ 1}}^{1} - \boldsymbol{P}_{i,n_{ 1} - 1}^{1} \bigr)\\ &\qquad {} + \bigl(10\gamma_{i,1} - 4\gamma_{i,1}n_{1} + n_{1} - n_{1}^{2}\bigr) \bigl( \boldsymbol{P}_{i,n_{ 1} - 1}^{1} - \boldsymbol{P}_{i,n_{ 1} - 2}^{1} \bigr) \\ &\quad = f^{2} \bigl[ \bigl(2\lambda_{2} + 4 \lambda_{2}m_{2} - m_{2} + m_{2}^{2} \bigr) \bigl(\boldsymbol{P}_{0,j}^{2} - \boldsymbol{P}_{1,j}^{2} \bigr)\\ &\qquad {} + \bigl(10\lambda_{2} - 4\lambda_{2}m_{2} + m_{2} - m_{2}^{2}\bigr) \bigl(\boldsymbol{P}_{1,j}^{2} - \boldsymbol{P}_{2,j}^{2}\bigr) \bigr] \\ &\quad (i = j = 0,1, \ldots,m_{1}). \end{aligned} $$

To sum up, if the two surfaces $\boldsymbol{S}_{1}(u,v;\lambda_{1},\gamma_{i,1})$ and $\boldsymbol{S}_{2}(u,v;\lambda_{2},\gamma_{i,2})$ satisfy both (20) and (23), the two surfaces reach G^2^ smooth continuity in the direction of *u* and *v* at the joint, and Theorem 2 gets proved. Obviously, if the shape parameters and control mesh points of the two surfaces satisfy (20), the two surfaces reach G^1^ smooth continuity in the direction of *u* and *v* at the joint. □

### Smooth continuity in the *v* direction

Similar to the G^2^ continuity conditions in the *u* direction between generalized Bézier-like surfaces, the following G^2^ continuity conditions in the *v* direction can be proved to be correct.

#### Theorem 3

*If the two adjacent generalized Bézier*-*like surfaces* $\boldsymbol{S}_{1}(u,v; \lambda_{1},\gamma_{i,1})$ *and* $\boldsymbol{S}_{2}(u,v; \lambda_{2},\gamma_{i,2})$ *satisfy all the following conditions*:

24 $$ \textstyle\begin{cases} n_{1} = n_{2}, \\ \boldsymbol{P}_{m_{1},j}^{1} = \boldsymbol{P}_{0,j}^{2}\quad (j = 0,1,\ldots,n_{1}), \\ \gamma_{m_{ 1} - 2,1} = \gamma_{m_{ 1} - 1,1} = \gamma_{m_{1},1} = \gamma_{0,2} = \gamma_{1,2} = \gamma_{2,2}, \\ \frac{\boldsymbol{P}_{m_{1},j}^{1} - \boldsymbol{P}_{m_{1} - 1,j}^{1}}{m_{2} + 2\lambda_{2}} = f\frac{\boldsymbol{P}_{1,j}^{2} - \boldsymbol{P}_{0,j}^{2}}{m_{1} + 2\lambda_{1}}\quad (j = 0,1, \ldots n_{1}), \\ (2\lambda_{1} + 4\lambda_{1}m_{1} - m_{1} + m_{1}^{2})(\boldsymbol{P}_{m_{ 1},j}^{1} - \boldsymbol{P}_{m_{ 1} - 1,j}^{1}) \\ \qquad {}+ (10\lambda_{1} - 4\lambda_{1}m_{1} + m_{1} - m_{1}^{2})(\boldsymbol{P}_{m_{ 1} - 1,j}^{1} - \boldsymbol{P}_{m_{ 1} - 2,j}^{1}) \\ \quad = f^{2} [ (2\lambda_{2} + 4\lambda_{2}m_{2} - m_{2} + m_{2}^{2})(\boldsymbol{P}_{0,j}^{2} - \boldsymbol{P}_{1,j}^{2})\\ \qquad {} + (10\lambda_{2} - 4\lambda_{2}m_{2} + m_{2} - m_{2}^{2})(\boldsymbol{P}_{1,j}^{2} - \boldsymbol{P}_{2,j}^{2}) ] \\ \quad (j = 0,1, \ldots,n_{1}) \end{cases} $$

*surfaces* $\boldsymbol{S}_{1}$ *and* $\boldsymbol{S}_{2}$ *reach G* ^2^ *smooth continuity in the* *v* *direction at the joint*, *where* $f > 0$ *is a real constant*.

#### Proof

The proof process of this theorem is similar to that of Theorem 1 and Theorem 2, so it is not covered here. □

Obviously, when all the shape parameters in $\mbox{Theorem~1} \sim \mbox{Theorem~3}$ are equal to 0, these continuity conditions above degrade into the corresponding G^2^ continuity conditions for high-order classical Bézier surfaces; when $\gamma_{0,1} = \gamma_{1,1} = \cdots = \gamma_{m_{1},1}$ and $\gamma_{0,2} = \gamma_{1,2} = \cdots = \gamma_{m,2}$, the continuity conditions in $\mbox{Theorem~1} \sim \mbox{Theorem~3}$ degrade into the corresponding G^2^ continuity conditions for Bézier-like surfaces in [17].

## Steps and examples of G^2^ smooth continuity between generalized Bézier-like surfaces

### Steps of G^2^ smooth continuity between generalized Bézier-like surfaces

Using the smooth continuity of generalized Bézier-like surfaces with shape adjustability, various complex surfaces can be designed handily and flexibly in engineering. In this section, we take the G^2^ smooth continuity in the *u* direction between two generalized Bézier-like surfaces as an example (the other two directions can be discussed similarly) to show the basic steps of G^2^ smooth continuity between generalized Bézier-like surfaces. On the basis of the conclusion in Theorem 1, the steps are as follows:

Step 1. According to designing requirement, give the order $m_{1}$, $n_{1}$ of the initial generalized Bézier-like surface $\boldsymbol{S}_{1}(u,v;\lambda_{1},\gamma_{i,1})$ and its control mesh points $\boldsymbol{P}_{i,j}^{1}$ ($i = 0,1, \ldots,m_{1}$; $j = 0,1, \ldots,n_{1}$) as well as shape parameters $\lambda_{1}$, $\gamma_{i,1}$.

Step 2. Let $m_{1} = m_{2}$, $\lambda_{1} = \lambda_{2}$ and $\boldsymbol{P}_{i,n_{1}}^{1} = \boldsymbol{P}_{i,0}^{2}$ ($i = 0,1, \ldots,m_{1}$), so that $\boldsymbol{S}_{1}(u,v;\lambda_{1},\gamma_{i,1})$ and $\boldsymbol{S}_{2}(u,v;\lambda_{2},\gamma_{i,2})$ possess a common boundary to reach G^0^ continuity.

Step 3. Give the values of the shape parameter $\gamma_{i,2}$ and the constant $f > 0$ as well as the other order $n_{2}$ of the second generalized Bézier-like surface $\boldsymbol{S}_{2}(u,v;\lambda_{2},\gamma_{i,2})$. On the basis of Step 2, calculate the second row control points $\boldsymbol{P}_{i,1}^{2}$ ($i = 0,1, \ldots,m_{1}$) of $\boldsymbol{S}_{2}(u,v;\lambda_{2},\gamma_{i,2})$ according to (11).

Step 4. On the basis of Step 2 and Step 3, calculate the third row control points $\boldsymbol{P}_{i,2}^{2}$ ($i = 0,1, \ldots,m_{1}$) of $\boldsymbol{S}_{2}(u,v;\lambda_{2},\gamma_{i,2})$ according to (18).

Step 5. Given the remaining $n_{2} - 2$ control points $\boldsymbol{P}_{i,j}^{2}$ ($i = 0,1, \ldots,m_{2}$; $j = 3,4, \ldots,n_{2}$) of $\boldsymbol{S}_{2}(u,v;\lambda_{2},\gamma_{i,2})$ freely, the G^2^ smooth continuity between two generalized Bézier-like surfaces in the *u* direction is achieved.

Repeating the steps above, G^2^ smooth continuity between multiple generalized Bézier-like surfaces will be achieved.

### Examples of G^2^ smooth continuity between generalized Bézier-like surfaces

**Figure 5:**
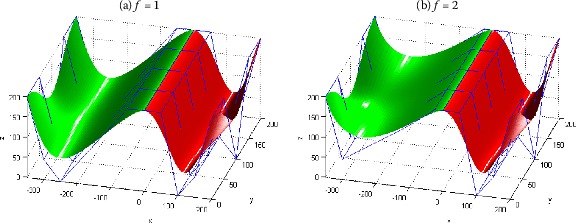
**G** ^**2**^ **continuity condition in the** ***v*** **direction between two adjacent generalized Bézier-like surfaces.**

In order to demonstrate the conclusions above, Figure 5 gives an example to show the G^2^ smooth continuity between two generalized Bézier-like surfaces of degree $(4, 4)$ in the *v* direction. In this figure, the red surface is the initial one $\boldsymbol{S}_{1}$; the green surface $\boldsymbol{S}_{2}$ is constructed according to the conclusion in Theorem 3, which reaches G^2^ smooth continuity with the red surface $\boldsymbol{S}_{1}$ in the *v* direction at the joint; Figures 5(a) and 5(b) show the graphs of the piecewise surface composed of $\boldsymbol{S}_{1}$ and $\boldsymbol{S}_{2}$ with the scaling factor *f* between their normal vectors equaling 1 and 2. To analyze the influence rule of *f* on the shape of the piecewise surface conveniently, all the shape parameters $\lambda_{1}$, $\lambda_{2}$, $\gamma_{i,j}$ ($i = 0,1,2,3,4$; $j = 1,2$) of the two piecewise surfaces in Figures 5(a) and 5(b) are the same and equal 1.

From the fourth and fifth equations of (24) and Figure 5, the scaling factor *f* between their normal vectors can be used to adjust the positions of the second and third row control points of the green surface $\boldsymbol{S}_{2}$. The bigger (or smaller) the value of *f* is, the closer (or farther away) the control points $\boldsymbol{P}_{i,1}^{2}$ (or $\boldsymbol{P}_{i,2}^{2}$) move to the control points $\boldsymbol{P}_{i,0}^{2}$ (or $\boldsymbol{P}_{i,1}^{2}$), where $\boldsymbol{P}_{i,0}^{2}$, $\boldsymbol{P}_{i,1}^{2}$, $\boldsymbol{P}_{i,2}^{2}$ ($i = 0,1,2,3,4$) are the first, second and third row control points of the green surface $\boldsymbol{S}_{2}$.

From the smooth continuity result in Figure 5, the piecewise generalized Bézier-like surface composed of $\boldsymbol{S}_{1}$ and $\boldsymbol{S}_{2}$ is smooth and continuous at the joint, so the result of smooth continuity is quite good, and thus can better satisfy actual needs.

## Shape adjustment of piecewise surfaces based on G^2^ smooth continuity

This section will focus on the shape adjustment of piecewise generalized Bézier-like surfaces with G^2^ smooth continuity. For simplicity, we take the smooth continuity between two generalized Bézier-like surfaces as an example to show the shape adjustment of piecewise surfaces. The situations for multiple surface patches can be discussed similarly, so they are not covered here. Compared with the smooth continuity between classical Bézier surfaces, the major advantage of the method in this paper is that apart from modifying control mesh points, we can also adjust the local or global shape of a piecewise surface by modifying its shape parameters with the overall smoothness of the surface remaining unchanged.

### Proposition 3

*For a piecewise generalized Bézier*-*like surface with G* ^2^ *smooth continuity*, *the following conclusions are established under the condition that the control mesh points and G* ^2^ *smooth continuity of the surface remain unchanged*.

- *For a piecewise generalized Bézier*-*like surface with G* ^2^ *smooth continuityin the u direction*, *we can adjust the global shape of the surface by changing the shape parameters* $\lambda_{1}$ *and* $\lambda_{2}$ *simultaneously*, *but we cannot adjust its local shape by changing its shape parameters*.
- *For a piecewise generalized Bézier*-*like surface with G* ^2^ *smooth continuity in the direction of* *u* *and* *v*, *we can adjust its global shape by changing the shape parameters* $\lambda_{1}$, $\gamma_{i,2}$ ($i = 0,1, \ldots,m_{2}$) *simultaneously*; *meanwhile we can also adjust its local shape by changing the shape parameters* $\gamma_{i,2}$ ($i = 3,4, \ldots,m_{2}$).
- *For a piecewise generalized Bézier*-*like surface with G* ^2^ *smooth continuity in the* *v* *direction*, *we can adjust its global shape by changing the shape parameters* $\gamma_{i,1}$ ($i = 0,1, \ldots,m_{1}$) *and* $\gamma_{i,2}$ ($i = 1,2, \ldots,m_{2}$); *meanwhile we can also adjust its local shape by changing the shape parameters* $\gamma_{i,1}$ ($i = 0,1, \ldots,m_{1} - 3$) *or* $\gamma_{i,2}$ ($i = 3,4, \ldots,m_{2}$).

### Proof

(a) According to the equation $\lambda_{1} = \lambda_{2}$ in (5), when we change the value of the parameter $\lambda_{1}$ to adjust the shape of the surface $\boldsymbol{S}_{1}$, the value of the parameter $\lambda_{2}$ will also change necessarily to maintain the G^2^ smooth continuity, so does the shape of the surface $\boldsymbol{S}_{2}$. Therefore we can adjust the global shape of the piecewise surface by changing the shape parameters $\lambda_{1}$ and $\lambda_{2}$ simultaneously.

We can rewrite the fourth equation of (5) as

25 $$ \boldsymbol{P}_{i,n_{1}}^{1} - \boldsymbol{P}_{i,n_{1} - 1}^{1} = c_{i}\bigl(\boldsymbol{P}_{i,1}^{2} - \boldsymbol{P}_{i,0}^{2}\bigr)\quad (i = 0,1, \ldots,m_{1}), $$

where $c_{i} = f\frac{n_{2} + 2\gamma_{i,2}}{n_{1} + 2\gamma_{i,1}}$ is a scaling factor. When the control mesh points of the piecewise surface are kept unchanged, the scaling factors $c_{i}$ ($i = 0,1, \ldots,m_{1}$) also will not change. So when we change the values of the shape parameters $\gamma_{i,1}$ ($i = 0,1, \ldots,m_{1}$) to adjust the shape of the surface $\boldsymbol{S}_{1}$, the values of the shape parameters $\gamma_{i,2}$ ($i = 0,1, \ldots,m_{2}$) need to change necessarily, and vice versa. However, the two sets of modified shape parameters may not satisfy the fourth and fifth equations of (5) simultaneously. In other words, it is hard for the piecewise surface to maintain G^2^ smooth continuity, thus the shape parameters $\gamma_{i,1}$ ($i = 0,1, \ldots,m_{1}$) and $\gamma_{i,2}$ ($i = 0,1, \ldots,m_{2}$) cannot be used to adjust the local and global shape of the piecewise surface.

(b) According to the equation $\lambda_{1} = \gamma_{0,2} = \gamma_{1,2} = \gamma_{2,2}$ in (19), when we change the value of the parameter $\lambda_{1}$ to adjust the shape of the surface $\boldsymbol{S}_{1}$, the value of the shape parameters $\gamma_{0,2}$, $\gamma_{1,2}$, $\gamma_{2,2}$ and the shape of the surface $\boldsymbol{S}_{2}$ need to change necessarily to maintain the G^2^ smooth continuity. So we can change the shape parameters $\lambda_{1}$ and $\gamma_{i,2}$ ($i = 0,1, \ldots,m_{2}$) simultaneously to adjust the global shape of the piecewise surface. Furthermore, as the constraint equations for G^2^ smooth continuity in (19) do not contain the shape parameters $\gamma_{i,2}$ ($i = 3,4, \ldots,m_{2}$), we can modify these parameters to adjust the shape of the surface $\boldsymbol{S}_{2}$ so as to realize the local shape adjustment of the piecewise surface. In addition, by the proving method of conclusion (1), it can be proved that the shape parameters $\lambda_{2}$, $\gamma_{i,1}$ ($i = 0,1, \ldots,m_{1}$) cannot be used to adjust the local or global shape of the piecewise surface.

(c) Obviously, conclusion (3) can be proved to be correct by the proving method of conclusion (1) and (2), so its proof is not covered here. □

**Figure 6:**
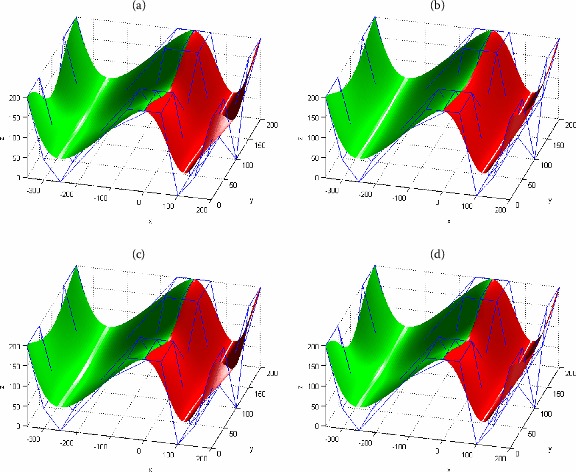
**Shape modification of piecewise generalized Bézier-like surfaces with G** ^**2**^ **continuity in the** ***v*** **direction.** **(a)** $\lambda _{1}= \lambda _{2} =1$, $\gamma _{i,1} = \gamma _{i,2} =1$ ($i=0,1,2,3,4$); **(b)** $\lambda _{1}= \lambda _{2} = 1$, $\gamma _{i,1} = \gamma _{i,2} = -1$ ($i=0,1,2,3,4$); **(c)** $\lambda _{1}= \lambda _{2} = 1$, $\gamma _{i,2} = -1$ ($i=3,4$), $\gamma _{0,2}= \gamma _{1,2}= \gamma _{2,2}= \gamma _{i,1} = 1$ ($i=0,1,2,3,4$); **(d)** $\lambda _{1}= \lambda _{2}=1$, $\gamma _{i,2}= \gamma _{4,1}= \gamma _{3,1}= \gamma _{2,1} = 1$ ($i=0,1,2,3,4$), $\gamma _{i,1} = -1$ ($i=0,1$).

Proposition 3 shows that piecewise generalized Bézier-like surfaces with G^2^ smooth continuity in the *v* direction have more free shape parameters independent of smooth continuity. Therefore the local shape adjustability of piecewise generalized Bézier-like surfaces with G^2^ smooth continuity in the *v* direction is superior to that in the other two directions. Figure 6 gives an example to show the local and global shape adjustment of a piecewise surface composed of two generalized Bézier-like surfaces of degree $(4, 4)$ with G^2^ smooth continuity in the *v* direction. In this figure, the shape parameters of the red surface $\boldsymbol{S}_{1}$ and the green surface $\boldsymbol{S}_{2}$ are marked as $\lambda_{1}$, $\gamma_{i,1}$ ($i = 0,1,2,3,4$) and $\lambda_{2}$, $\gamma_{i,2}$ ($i = 0,1,2,3,4$), respectively. Figure 6(a) shows the graph of the initial piecewise surface; Figures 6(c) and 6(d) show the graphs of the locally modified piecewise surface with the shape parameters $\gamma_{3,2}$, $\gamma_{4,2}$ and $\gamma_{0,1}$, $\gamma_{1,1}$ taking different values ($\gamma_{3,2}$, $\gamma_{4,2}$ and $\gamma_{0,1}$, $\gamma_{1,1}$ adjust the shape of the green surface $\boldsymbol{S}_{2}$ and the red surface $\boldsymbol{S}_{1}$, respectively); Figure 6(b) shows the graph of the globally modified piecewise surface with the shape parameters $\gamma_{i,j}$ ($i = 0,1,2,3,4$; $j = 1,2$) taking different values ($\gamma_{i,j}$ ($i = 0,1,2,3,4$; $j = 1,2$) adjust both shapes of $\boldsymbol{S}_{1}$ and $\boldsymbol{S}_{2}$).

## Conclusions

In this paper, we constructed a kind of generalized Bézier-like surfaces associated with multiple shape parameters. Then the G^2^ continuity conditions for the generalized Bézier-like surfaces of degree $(m, n)$ are derived, and the influence rules of the shape parameters on splicing surfaces are analyzed. We feel our work is significant since our proposals help to simplify the construction and computer realization of complex surfaces as well as extend the conclusions of [17]. The modeling examples showed the effectiveness of the proposed methods: the generalized Bézier-like surfaces have more powerful shape adjustability and approximation ability than the existing Bézier-like surfaces described in [17]. The advantages and features of the proposed methods can be summarized as follows:

- The proposed generalized Bézier-like surfaces of degree $(m, n)$ extend the conclusions of the Bézier-like surfaces given in [17].
- For piecewise generalized Bézier-like surfaces with G^2^ smooth continuity, we can adjust their global and local shape by changing their shape parameters.
- The G^2^ smooth continuity proposed in this paper is not only intuitive and easy to implement, but also offers more degrees of freedom for constructing complex surfaces in engineering design.

It is worth noting that the proposed methods in this paper are the first to consider G^2^ geometric continuity conditions for the generalized Bézier-like surfaces of degree $(m, n)$.
